# CAFNet-DG: Task-Specific Modeling and Prevalence-Aware Evaluation for Adverse Drug Reaction Prioritization with Ordinal-Frequency Estimation

**DOI:** 10.3390/ijms27198685

**Published:** 2026-09-28

**Authors:** Wen Li, Zhiyu Xu, Xiangqiong Wu, Ju Xiang, Xiaojie Zhang

**Affiliations:** 1 College of Electronic Information, Hunan First Normal University, Changsha 410205, China; wenli@hnu.edu.cn (W.L.); 13454048968@163.com (Z.X.); wuxiangqiong@hnu.edu.cn (X.W.); 2 School of Computer Science and Technology, Changsha University of Science and Technology, Changsha 410114, China; xiang.ju@foxmail.com

**Keywords:** adverse drug reaction, side-effect prioritization, annotation prevalence, molecular graph, graph neural network, ordinal-frequency estimation, drug-disjoint prediction

## Abstract

Adverse drug reaction (ADR) prioritization and ordinal-frequency estimation are related but distinct prediction objectives, while aggregate ranking performance in sparse annotation matrices can be strongly influenced by frequently annotated side effects. Within CAFNet-DG, CAFNet-D uses task-specific association and ordinal-frequency functions built on shared molecular–relational representations, while annotation prevalence is evaluated explicitly within each training fold. On a SIDER-derived dataset comprising 750 drugs and 994 side effects, replacing a shared output with task-specific functions produced the largest joint change in staged drug-disjoint ablation, increasing mAP from 0.340 to 0.382 and reducing RMSE from 1.754 to 1.142. A prevalence-only ranking achieved an mAP of 0.410, close to the CAFNet-DG value of 0.413, indicating that aggregate ADR ranking is strongly associated with the benchmark annotation distribution. Prevalence-matched, prevalence-stratified, frequently annotated side-effect removal, and per-drug analyses were therefore used to distinguish drug-conditioned ranking variation from global annotation-prevalence effects. An equal-weight same-architecture ensemble control yielded no significant differences from CAFNet-DG across seven ranking metrics after Holm correction; generic variance reduction therefore could not be excluded as an explanation for the modest score-integration gain. Scaffold-disjoint and independent-data evaluations showed modest transfer, while ordinal-frequency agreement on CT-ADE remained limited. The results support the CAFNet-D task-specific formulation and prevalence-aware evaluation, while CAFNet-DG provides the score-integration setting examined under matched ensemble controls.

## 1. Introduction

Adverse drug reactions (ADRs) remain a major challenge to safe and effective pharmacotherapy. A meta-analysis of prospective hospital studies documented their substantial contribution to serious morbidity and mortality [1], and post-marketing surveillance has identified adverse drug events as an important factor in the withdrawal of approved medicines [2]. Pharmacovigilance draws on spontaneous reports, electronic health records, drug labels, clinical studies, and other real-world sources, while data-mining methods have expanded the capacity to identify candidate safety signals [3,4]. However, coverage across these sources remains incomplete, and reporting and observation biases complicate the identification of uncommon, delayed, or previously unrecognized events [3]. Computational models complement these sources by prioritizing plausible drug–side-effect associations for subsequent clinical, pharmacovigilance, or experimental assessment.

Computational ADR modeling encompasses two related objectives: association prediction and frequency estimation. The Side Effect Resource (SIDER) is a curated database of adverse drug reactions derived primarily from marketed-drug labels and related public documents. In addition to drug–side-effect associations, SIDER provides reported frequency categories where such information is available, making it relevant to both ADR prioritization and ordinal-frequency estimation [5]. Galeano et al. formulated side-effect frequency prediction from these ordered labels [6], and Zhao et al. introduced graph-attention-based multi-view learning for this task [7]. Subsequent studies have extended ADR occurrence and frequency modeling through hybrid multimodal fusion [8], Transformer-based cross-feature learning [9], asymmetric multi-task architectures [10], cross-attention-based interaction modeling [11], and early-stage drug-discovery information [12]. Relational learning methods have also been developed to improve ADR prediction for previously unseen drugs [13]. These studies have expanded drug and side-effect representations, but candidate ordering is generally based on pointwise association or frequency scores rather than treated as a distinct optimization objective.

ADR prioritization and ordinal-frequency estimation serve distinct purposes. For a given drug, prioritization orders candidate adverse events for subsequent assessment, whereas frequency estimation assigns a reported ordinal category to an observed drug–side-effect association. Candidate ordering is consequential in screening settings because only a subset of associations can be examined in detail. Pairwise and listwise learning-to-rank objectives directly model relative candidate order and complement pointwise prediction [14,15]. Pointwise association scores do not explicitly optimize within-drug ordering. A shared prediction function also couples ordering and ordinal estimation, although these objectives need not favor the same score function. These distinctions motivate task-specific association and ordinal-frequency functions while retaining frequency information in the final prioritization score.

The sparse, imbalanced SIDER-derived frequency matrix raises a separate evaluation concern. Item-popularity effects are well documented in ranking and recommender-system research, where popular items may dominate recommendation lists while long-tail items receive limited exposure [16,17,18]. Spontaneous pharmacovigilance reports are also subject to systematic reporting biases, including changes following safety alerts and differences associated with event notoriety or seriousness [19,20]. Although these effects are documented outside the SIDER setting, they warrant direct examination of whether non-uniform annotation frequencies influence ADR ranking. Side effects in the SIDER-derived matrix differ markedly in the number of drugs for which a label is observed [6]. This count, defined within each training fold, is termed *side-effect annotation prevalence*; it characterizes the benchmark annotation distribution rather than population-level ADR incidence. A prevalence-only ranking assigns the same ordering of side effects to every drug but can perform well on aggregate retrieval measures when commonly annotated events recur across drugs. High mAP or nDCG is therefore compatible with either drug-conditioned ranking variation or the global prevalence structure of the annotation matrix. This ambiguity motivates assessment of whether a learned score adds drug-specific ranking variation after controlling for annotation prevalence.

Molecular and side-effect relational representations may support such drug-conditioned variation. Molecular graphs encode atom-level chemical structure, whereas the side-effect relation graph encodes similarities among frequency profiles observed in the training data. Graph neural networks and attention mechanisms learn structural and relational representations, and cross-attention couples them at the drug–side-effect level [9,11,21,22]. Recent models have also incorporated biological, semantic, or early-stage drug-development modalities [8,12], while informative relational learning has been used to address generalization to novel drugs [13]. Because these auxiliary modalities are not uniformly available for all drugs in the fixed SIDER benchmark, this study holds benchmark inputs and data partitions constant when evaluating score integration and explicitly controls for annotation prevalence.

CAFNet-DG was developed to study ADR prioritization and ordinal-frequency estimation under sparse and prevalence-skewed supervision. Within the framework, CAFNet-D uses distinct prediction functions for association-oriented prioritization and ordinal-frequency estimation while retaining frequency information in the prioritization score. Side-effect annotation prevalence is examined as a benchmark-distribution factor through prevalence-only ranking, matched-prevalence controls, prevalence-stratified analyses, removal of frequently annotated side effects, and per-drug comparisons. CAFNet-DG then combines the CAFNet-D prioritization score with an independently fitted CAFNet graph-interaction score. An equal-weight same-architecture ensemble serves as the primary control for this integration step, while scaffold-disjoint and independent-data analyses assess structural and cross-source transfer.

## 2. Results

### 2.1. Task-Specific Formulation and ADR Prioritization Performance

**Figure 1:**
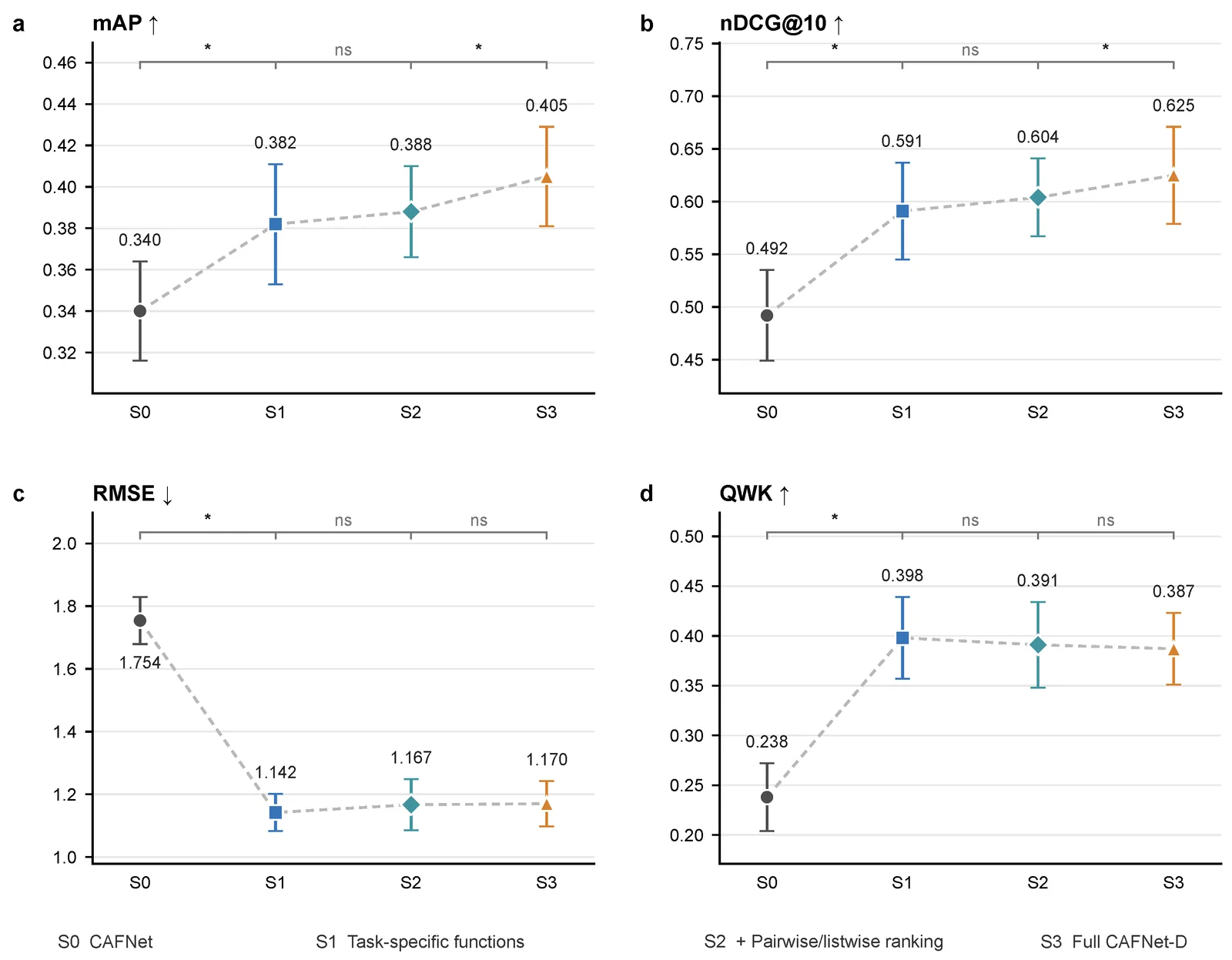
**Staged ablation under 10-fold drug-disjoint evaluation.** Points and error bars denote the mean ± standard deviation across the 10 folds for (**a**) mean average precision (mAP), (**b**) normalized discounted cumulative gain at 10 (nDCG@10), (**c**) root mean squared error (RMSE), and (**d**) quadratic-weighted kappa (QWK). S0 denotes CAFNet with a shared output; S1 replaces this output with task-specific association and ordinal-frequency functions; S2 adds pairwise and listwise ranking supervision; and S3 denotes the complete CAFNet-D formulation with drug- and side-effect-specific bias terms and the training-fold annotation-prevalence term. Brackets denote adjacent-stage comparisons based on two-sided Wilcoxon signed-rank tests with Holm adjustment within the ranking and ordinal metric families; an asterisk denotes adjusted p<0.05, and ns denotes adjusted p≥0.05. Exact adjusted *p*-values are reported in Supplementary Table S5. Each variant was fitted independently using the same partitions. Connecting lines denote only the pre-specified ablation order. Axis ranges are metric-specific; visual slopes should therefore be compared only within each panel. ↑—increase, ↓—decrease.

The staged drug-disjoint ablation of CAFNet-D first examined whether using distinct prediction functions for ADR prioritization and ordinal-frequency estimation improved the two objectives relative to the shared-output formulation. Replacing the shared output with task-specific association and ordinal-frequency functions produced the largest joint change in the ablation sequence (Figure 1). From S0 to S1, mAP increased from 0.340 to 0.382, nDCG@10 increased from 0.492 to 0.591, RMSE decreased from 1.754 to 1.142, and QWK increased from 0.238 to 0.398. All four comparisons remained significant after Holm adjustment. Adding pairwise and listwise supervision in S2 produced smaller numerical changes, none of which remained significant after within-family adjustment. Adding the bias and annotation-prevalence terms in S3 increased mAP from 0.388 to 0.405 and nDCG@10 from 0.604 to 0.625, whereas RMSE and QWK remained statistically unchanged. This pattern is consistent with the model formulation: S1 introduces the task-specific functions responsible for the main joint improvement, whereas S3 adds bias terms to the ordinal-frequency estimate and an annotation-prevalence term to the prioritization score. In the staged analysis, the additional statistically significant changes were confined to the ranking measures. Complete results and adjusted *p*-values are reported in Supplementary Tables S4 and S5.

Among models evaluated with matched inputs and partitions, CAFNet-DG attained the highest mean values across all seven ranking measures under warm-start evaluation (Table 1). Under drug-disjoint evaluation, CAFNet-DG attained the highest mean values for mAP, AUROC, AUPR, nDCG@10, and P@1, whereas CAFNet-D attained marginally higher mean values for P@15 and R@15.

CAFNet-DG attained higher means than DSGAT on all seven ranking measures. Six differences remained significant after Holm adjustment, whereas AUROC differed only numerically (0.826 versus 0.822). With ρ=0.6, CAFNet-DG yielded higher fold-level mAP and AUROC than CAFNet-D after Holm adjustment (Holm-adjusted Wilcoxon p=0.0234 and p=0.0137, respectively). Differences in AUPR, nDCG@10, and P@1 were non-significant; P@15 and R@15 were marginally lower and likewise non-significant. Because ρ=0.6 was retained after post hoc examination of the same benchmark-fold predictions, these single-fit comparisons are conditional on that coefficient. Section 2.3 provides the corresponding same-architecture ensemble comparison.

Omitting the bias and annotation-prevalence terms reduced top-ranked retrieval, whereas omission of the association-specific term produced only small numerical changes under the same drug-disjoint protocol. Supplementary Table S3 reports the component-removal results; Supplementary Tables S4 and S5 report the complete staged results and Holm-adjusted paired comparisons, respectively.

### 2.2. Influence of Side-Effect Annotation Prevalence

**Figure 2:**
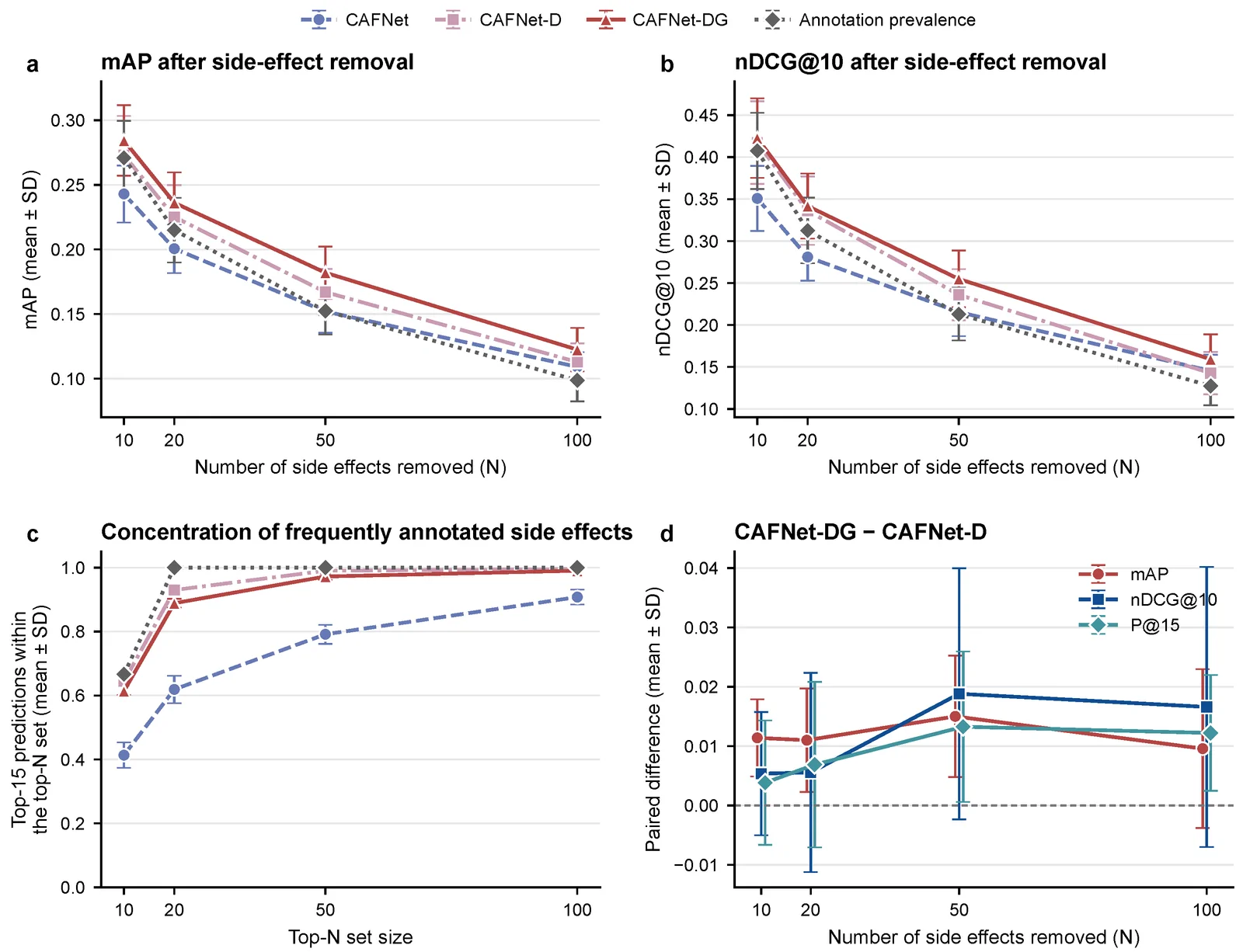
**Ranking after prevalence-based removal of frequently annotated side effects.** Within each training fold, side effects were ordered by the number of drugs with a positive annotation. The top-*N* side effects were then removed before the ranking metrics were recalculated. Panels (**a**,**b**) report mean average precision (mAP) and normalized discounted cumulative gain at 10 (nDCG@10), respectively. All non-zero frequency labels were treated as binary relevance. Metrics were calculated only for held-out drugs retaining at least one positive label. Before removal, panel (**c**) reports the proportion of each model’s top-15 predictions that belonged to the top-*N* side effects by annotation prevalence. Annotation prevalence is included as a non-personalized reference ranking. Panel (**d**) reports fold-wise paired differences between CAFNet-DG and CAFNet-D for mAP, nDCG@10, and precision at 15 (P@15). Markers in panel (**d**) are offset slightly along the horizontal axis for visibility. Points and error bars denote the mean ± standard deviation across the 10 drug-disjoint folds. Panel (**d**) is displayed descriptively without significance symbols; the corresponding two-sided paired Wilcoxon tests and Holm-adjusted *p*-values are reported in the Supplementary analysis accompanying Table S9.

The non-personalized annotation-prevalence ranking attained an mAP of 0.410, close to the CAFNet-DG value of 0.413 (Table 2). This proximity is consistent with a strong association between aggregate mAP and annotation prevalence. The top-15 predictions from CAFNet-D and CAFNet-DG remained concentrated among the most frequently annotated side effects, whereas those from CAFNet were less concentrated (Figure 2c).

With progressive removal of the most frequently annotated side effects, mAP and nDCG@10 decreased as the removal threshold increased (Figure 2a,b). At each threshold, the mean difference between CAFNet-DG and CAFNet-D remained positive; however, fold-wise variability was substantial, and no consistent advantage emerged for low-prevalence ADRs (Figure 2d).

Relative to CAFNet, the prevalence-enriched CAFNet-D score improved aggregate ranking but yielded lower AP in the low- and middle-prevalence strata. CAFNet-DG showed higher AP than the single CAFNet-D fit in both strata and a higher matched-control AUPR. Relative to that single CAFNet-D fit, CAFNet-DG increased AP for 59.5% of the 750 held-out drugs. These single-fit differences characterize ranking variation and are interpreted alongside the ensemble control in Section 2.3. Among drugs evaluable for the corresponding analyses, the proportions with positive differences favoring CAFNet-DG were 57.5% for low-prevalence AP, 60.9% for middle-prevalence AP, and 64.1% after removal of the 100 most frequently annotated side effects. Wilson 95% confidence intervals and two-sided exact binomial tests against a 50% directional null are reported in Supplementary Table S8.

### 2.3. Same-Architecture Ensemble Control for Score Integration

To assess whether the modest gain associated with CAFNet-DG could be distinguished from the generic effect of averaging independently fitted predictors, we compared the reference CAFNet-DG model with an equal-weight same-architecture CAFNet-D ensemble under the identical 10 drug-disjoint folds. The reference model used $0.6D_{3}+0.4C_{\mathrm{ref}}$, where $D_{3}$ denotes the CAFNet-D fit used in the reported CAFNet-DG analysis and $C_{\mathrm{ref}}$ denotes the independently fitted CAFNet score source paired with it. The control, denoted D-Ens50, used $0.5D_{3}+0.5D_{42}$, where $D_{42}$ denotes an independently fitted CAFNet-D model Table 3.

CAFNet-DG was numerically higher on five metrics, whereas D-Ens50 was higher on P@15 and R@15; none of the seven paired differences remained significant after Holm correction. The control therefore did not show an aggregate advantage over same-architecture ensembling. Additional ensemble sensitivity analyses are reported in Supplementary File S1.

### 2.4. Ordinal-Frequency Estimation

CAFNet-D and CAFNet-DG share the same ordinal-frequency output, which achieved the best mean performance across all five ordinal measures under both warm-start and drug-disjoint evaluation (Table 4).

Ordinal agreement was stronger under warm-start than under drug-disjoint evaluation: QWK decreased from 0.640 to 0.377, and within-one-class accuracy decreased from 0.956 to 0.841. These reductions indicate weaker ordinal generalization beyond the training drug set.

### 2.5. Scaffold-Disjoint Evaluation

No scaffold group or exact canonical structure was shared between the training and test sets. CAFNet-DG attained an mAP of 0.409±0.020, compared with 0.404±0.021 for CAFNet-D and 0.334±0.013 for CAFNet. After the 100 most frequently annotated side effects were removed, the respective mAP values were 0.117±0.014, 0.104±0.016, and 0.105±0.015. Differences between CAFNet-DG and CAFNet were non-significant in the low- and middle-prevalence strata. A small subset of test drugs nevertheless had highly similar analogues in the training set. Partition details and the full scaffold-disjoint comparison are reported in Supplementary Table S11.

### 2.6. Evaluation on Independent Data

The CT-ADE-PT mapping comprised 1336 non-SIDER adverse-event pairs involving 29 drugs and 27,538 candidate background pairs. CAFNet-DG attained the highest pooled AUROC and AUPR (Table 5). Per-drug Wilcoxon tests showed significantly higher values for CAFNet-DG than for CAFNet on both measures after Holm adjustment; comparisons with CAFNet-D and the annotation-prevalence control were non-significant. In the same-drug prevalence-matched analysis, CAFNet-DG attained AUROC and AUPR values of 0.679 and 0.321, respectively; comparisons with CAFNet and CAFNet-D were non-significant.

Ordinal-frequency generalization to CT-ADE was limited. Across 2781 non-SIDER frequency pairs, the uncalibrated CAFNet-D estimates had significantly higher RMSE and MAE and lower within-one-class accuracy than the training-side-effect mean baseline; differences in QWK and Spearman correlation were non-significant. Isotonic calibration reduced RMSE and MAE to 0.867 and 0.697, respectively, but per-drug comparisons with the side-effect mean remained non-significant; QWK decreased to 0.209. Full calibration results are provided in Supplementary Table S1.

After pairs overlapping with SIDER were excluded, the OFFSIDES evaluation included 35,683 mapped positive pairs involving 463 drugs. CAFNet-DG yielded pooled AUROC and AUPR values of 0.5183 and 0.5175, respectively, compared with 0.5023 and 0.5058 for the annotation-prevalence control. Per-drug comparisons with CAFNet-D yielded statistically detectable but small differences: mean AUROC was 0.5218 versus 0.5205, and mean AUPR was 0.5307 versus 0.5291.

The OnSIDES and FDA evaluations included 91 and 53 mapped positive pairs, respectively, after pairs overlapping with SIDER were excluded. Neither evaluation identified a significant difference between CAFNet-DG and either CAFNet or CAFNet-D. Details of the independent-data analyses are reported in Supplementary Table S1.

## 3. Discussion

### 3.1. Interpretation and Implications

In the staged ablation, replacing the shared output with task-specific association and ordinal-frequency functions produced the largest joint changes in ranking and ordinal-frequency measures. Pairwise and listwise ranking supervision produced smaller changes, whereas the bias and annotation-prevalence terms primarily affected aggregate retrieval. The staged pattern therefore supports the CAFNet-D task-specific output formulation, rather than score integration, as the strongest method-level contribution of the present experiments. This formulation differs from earlier ADR models focused on binary association prediction or pointwise frequency regression [6,7,8].

Annotation prevalence was strongly associated with aggregate drug-disjoint performance. The non-personalized annotation-prevalence control attained an mAP close to that of CAFNet-DG, and removing the most frequently annotated side effects reduced performance across all models. In prevalence-matched, prevalence-stratified, and per-drug analyses, learned scores changed individual rankings, but the direction and magnitude of these changes varied among drugs and did not consistently favor low-prevalence ADRs. Aggregate retrieval measures should therefore be interpreted jointly with prevalence-controlled analyses rather than as direct evidence of drug-specific signal recovery.

The equal-weight same-architecture control did not show a ranking advantage for score integration beyond same-architecture averaging. CAFNet-DG is therefore interpreted as combining a prevalence-enriched score with an independently fitted graph-interaction score, without assigning the small aggregate gain to either score source.

The graph-interaction score incorporates molecular structure, side-effect relations, and cross-attention-derived information; it does not directly represent molecular mechanism. Under scaffold-disjoint evaluation, CAFNet-DG yielded only a small numerical mAP advantage over CAFNet-D, and several test drugs had highly similar analogues in the training data. On independent data, differences relative to CAFNet-D and the annotation-prevalence controls were small or statistically non-significant, with the exception of statistically detectable but small OFFSIDES differences. These results support use of the model for candidate prioritization rather than mechanistic or clinical inference.

Compound–target affinity could provide additional mechanistic information for ADR prioritization by representing intended and off-target interactions that are not directly encoded by molecular structure and side-effect co-annotation patterns. Its relationship with toxicity, however, cannot be reduced to an inverse association between affinity and administered dose. Toxic effects also depend on target selectivity, systemic exposure, pharmacokinetics, metabolism, tissue distribution, and target engagement. Future work could evaluate affinity and exposure descriptors as additional modalities under availability-matched and prevalence-controlled protocols.

### 3.2. Limitations and Future Directions

The external data sources differed in study design and evidentiary status and lacked adjudicated causal labels and confirmed negative pairs. Isotonic calibration reduced scale error, but ordinal agreement on CT-ADE remained low. Sensitivity analysis indicated that ordinal errors varied with the target (ϵ=0.5) and weight $\left(\lambda_{0}=0.03\right)$ assigned to unobserved SIDER entries. Both hyperparameters were fixed without nested validation.

The same SIDER-derived benchmark folds were examined during model development and exploratory analysis; ρ=0.6 was retained after a post hoc sweep of stored drug-disjoint predictions and then applied uniformly, without fold-specific tuning, checkpoint selection, or early stopping. The resulting tests do not account for adaptive model development and may therefore be optimistic. The primary equal-weight control does not exhaust possible ensemble baselines; broader sensitivity analyses are reported in Supplementary Table S2.

The ranking evaluation treated all non-zero frequency labels as a single relevance class. Score integration depends on the scaling of its component scores and the choice of ρ; reverse cross-attention also exhibits minor mini-batch dependence. Future work should examine nested selection of frequency-regularization and integration parameters, batch-independent interaction functions, and evaluation in temporally separated or electronic health record cohorts.

## 4. Materials and Methods

### 4.1. Dataset and Problem Formulation

This study used a dataset derived from version 4.1 of the SIDER database [5], a resource widely used in drug–side-effect frequency prediction studies. The dataset comprises 750 drugs, 994 side effects, and 37,071 annotated drug–side-effect pairs.

Reported side-effect frequencies are encoded as ordered integer labels ranging from 1 to 5, with higher values corresponding to higher frequency categories. Labels 1 and 5 denote the lowest and highest reported categories, respectively. Pairs lacking reported frequency information are assigned a value of 0, which denotes unknown or unreported frequency rather than a reported ordinal category or confirmed absence.

Let $D={\left\{d_{i}\right\}}_{i=1}^{750}$ and $E={\left\{e_{j}\right\}}_{j=1}^{994}$ denote the drug and side-effect sets, respectively. Their observed relations are represented by the sparse ordinal matrix $S\in R^{750\times 994}$, in which 4.97% of the entries are non-zero. The frequency-label distribution is summarized in Table 6.

For each pair $\left(d_{i},e_{j}\right)$, let $y_{ij}=I\left[S_{ij}>0\right]$ denote binary relevance. Evaluation considers two task-level outputs: a prioritization score for ranking candidate side effects for each drug and a scalar estimate of the ordinal label for an observed association. The model additionally learns an association logit as a supervised internal component of the prioritization score. Side-effect annotation prevalence, denoted by $p_{e_{j}}$, is defined as the number of drugs with a non-zero label for $e_{j}$ within a training fold and characterizes the fold-specific benchmark annotation distribution. The ordinal output estimates the reported SIDER frequency category for each observed association and does not represent patient-level incidence.

### 4.2. Molecular–Relational Representation Learning

**Figure 3:**
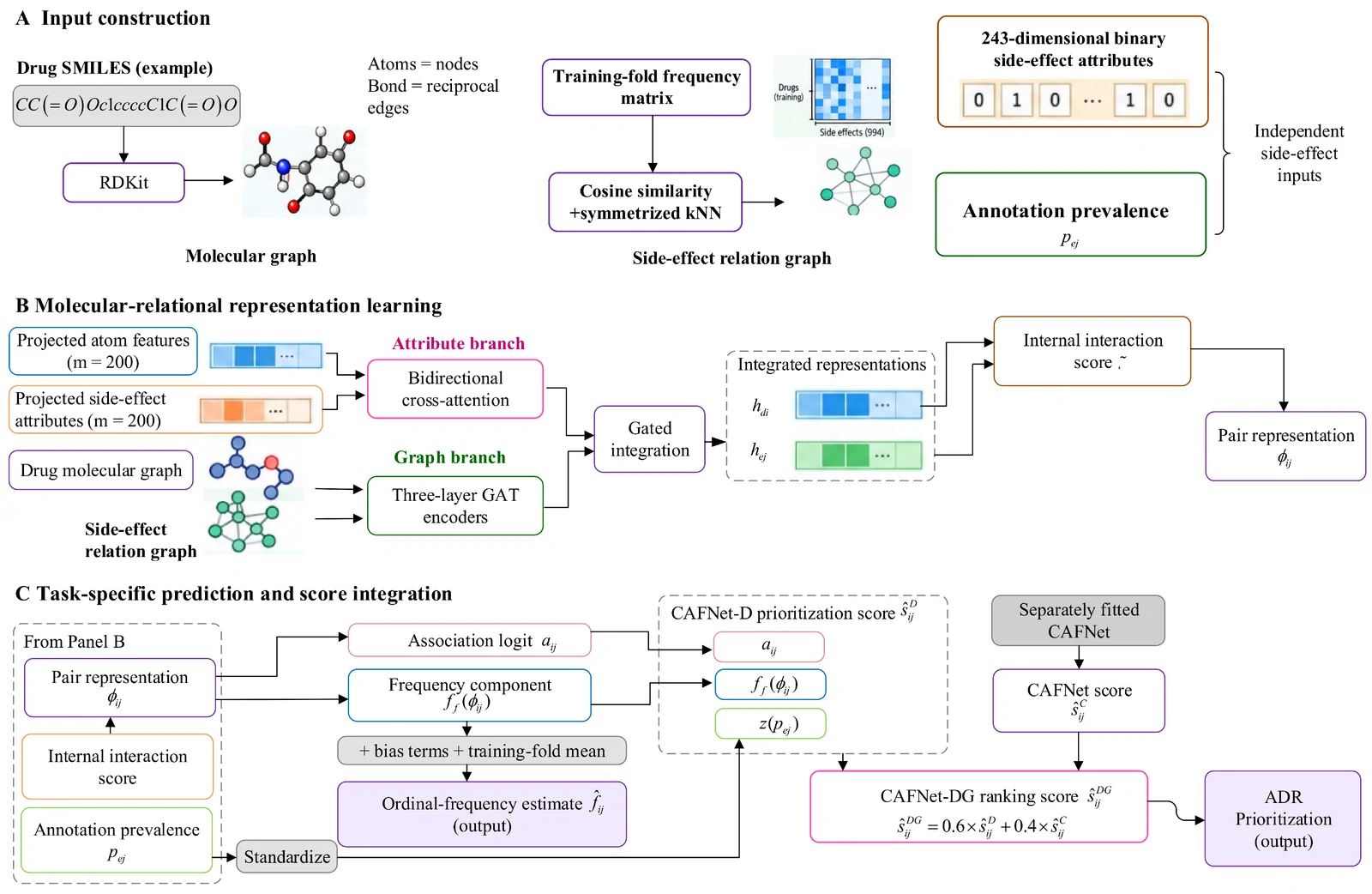
**CAFNet-DG workflow.** (**A**) Drug SMILES strings are converted to molecular graphs with RDKit, while the side-effect relation graph and annotation prevalence are derived exclusively from the training-fold frequency matrix; benchmark-provided 243-dimensional side-effect attributes are retained unchanged. (**B**) Attribute-based cross-attention and graph-attention branches are fused to obtain drug and side-effect representations for task-specific association and ordinal-frequency prediction. (**C**) CAFNet-D and CAFNet are fitted separately; their ranking scores are integrated for ADR prioritization, whereas the ordinal-frequency estimate is taken directly from CAFNet-D.

CAFNet-D and CAFNet use the same molecular–relational representation backbone design but differ in their output layers and training objectives. They are instantiated and fitted separately within each data partition. In each model, drug molecular graphs, a training-fold-specific side-effect relation graph, and benchmark-provided side-effect attributes are mapped to latent drug and side-effect representations. CAFNet-D produces the prioritization and ordinal-frequency outputs, whereas CAFNet provides the graph-interaction score. Figure 3 summarizes the mapping from the inputs to the model outputs and their subsequent score integration.

Each drug $d_{i}$ is represented as a molecular graph whose nodes correspond to atoms and whose undirected chemical bonds are encoded as pairs of reciprocal directed edges. Molecular graphs are constructed from SMILES strings using RDKit [23]. Atom attributes include atom type, degree, hydrogen count, valence, formal charge, hybridization, and aromaticity. Each side effect corresponds to a node in the relation graph and is associated with a benchmark-provided 243-dimensional binary attribute vector. Two complementary latent representations are constructed. The attribute-based representation couples projected atom and side-effect features through bidirectional dot-product attention, whereas three-layer graph-attention encoders produce graph-based representations [22]. Gated integration subsequently combines the attribute- and graph-based representations for each entity type [24].

Within each fold, the side-effect graph was reconstructed exclusively from the corresponding training frequency matrix using a symmetrized cosine *k*-nearest-neighbor rule; benchmark-provided side-effect attributes were retained unchanged. Fold-specific reconstruction prevented held-out entries from contributing to graph construction. However, because the graph was derived from the sparse training matrix, its topology could retain co-annotation and annotation-prevalence structure even though cosine similarity normalized the vector magnitudes.

Let $X_{B}^{a}\in R^{N_{B}\times m}$ denote the projected atom features of all drugs in the current mini-batch B, where $N_{B}$ is the total number of atoms across these drugs. Let $E^{a}\in R^{994\times m}$ denote the projected side-effect attribute features. The 109-dimensional atom descriptors and the 243-dimensional side-effect attribute vectors are independently mapped to the shared *m*-dimensional space by separate fully connected layers followed by ReLU activation. ReLU is retained as part of the common CAFNet representation backbone and is held constant across the matched CAFNet-family comparisons. Alternative activation functions were not evaluated in the present study. Bidirectional cross-attention is then applied to the projected node features:

(1) $$\begin{array}{cc} X_{B}^{c} & =g_{o,d}\left[\mathrm{softmax}\left(\frac{Q_{d}K_{e}^{⊤}}{\tau }\right)V_{e}\right],\\ E_{B}^{c} & =g_{o,e}\left[\mathrm{softmax}\left(\frac{Q_{e}K_{d,B}^{⊤}}{\tau }\right)V_{d,B}\right]. \end{array}$$

Drug-atom queries attend to side-effect keys and values, whereas side-effect queries attend to the projected atom features of the drugs in B. Direction-specific learned linear maps generate the query, key, and value matrices; $g_{o,d}$ and $g_{o,e}$ denote the corresponding learned affine output projections. The projected dimension was m=200, and the scaling denominator was fixed at τ=5 in all experiments.

For drug $d_{i}$, the attribute-based representation is computed by adding each projected atom feature to its corresponding cross-attention output and applying global max pooling over the atoms in the molecular graph:

(2) $$h_{d_{i}}^{\mathrm{att}}={\mathrm{MaxPool}}_{v\in V(d_{i})}\left(x_{iv}^{a}+x_{iv}^{c}\right).$$

For side effect $e_{j}$, the attribute-based representation is computed at the node level and therefore requires no graph-level pooling:

(3) $$h_{e_{j}}^{\mathrm{att}}=e_{j}^{a}+e_{j,B}^{c},\;j=1,\ldots ,994.$$

In parallel, three-layer graph-attention (GAT) encoders map the drug molecular graphs and the side-effect relation graph to graph-based representations. For each drug, the final atom embeddings are aggregated by global max pooling over its molecular graph:

(4) $$h_{d_{i}}^{g}={\mathrm{MaxPool}}_{v\in V(d_{i})}z_{iv}^{\left(3\right)}.$$

For each node in the side-effect relation graph, the final GAT output is retained directly, without graph-level pooling:

(5) $$h_{e_{j}}^{g}=z_{e_{j}}^{\left(3\right)},\;j=1,\ldots ,994.$$

The attribute-based and graph-based representations are then combined through gated feature integration. For drug $d_{i}$, the gating vector is

(6) $$g_{d_{i}}=\sigma \left(\mathrm{MLP}\left([h_{d_{i}}^{g},h_{d_{i}}^{\mathrm{att}}]\right)\right),$$

and the integrated drug representation is

(7) $$h_{d_{i}}=W_{d}\left[h_{d_{i}}^{g},h_{d_{i}}^{\mathrm{att}}\right]+\frac{1}{2}\left\{g_{d_{i}}⊙h_{d_{i}}^{g}+\left(1-g_{d_{i}}\right)⊙h_{d_{i}}^{\mathrm{att}}\right\}.$$

For each side-effect node, the gating vector is computed independently as

(8) $$g_{e_{j}}=\sigma \left(\mathrm{MLP}\left([h_{e_{j}}^{g},h_{e_{j}}^{\mathrm{att}}]\right)\right),$$

and the integrated side-effect representation is

(9) $$h_{e_{j}}=W_{e}\left[h_{e_{j}}^{g},h_{e_{j}}^{\mathrm{att}}\right]+\frac{1}{2}\left\{g_{e_{j}}⊙h_{e_{j}}^{g}+\left(1-g_{e_{j}}\right)⊙h_{e_{j}}^{\mathrm{att}}\right\}.$$

Within CAFNet-D, the inner product of the drug and side-effect representations defines an internal interaction score:

(10) $${\tilde{s}}_{ij}^{D,0}=h_{d_{i}}^{⊤}h_{e_{j}}.$$

For the independently fitted CAFNet model, the graph-interaction score is defined as the raw inner product of its drug and side-effect representations:

(11) $${\hat{s}}_{ij}^{C}={\left(h_{d_{i}}^{C}\right)}^{⊤}h_{e_{j}}^{C}.$$

Both interaction scores are derived from molecular structure and side-effect relations, with cross-attention coupling the drug and side-effect representations; the two models use distinct parameter sets. The reverse-attention representation $E_{B}^{c}$ depends on the composition of the drug mini-batch B.

### 4.3. Task-Specific Prediction Model

CAFNet-D uses task-specific functions for association prediction and ordinal-frequency estimation. The association logit is supervised by the binary relevance indicator and contributes to the final prioritization score, whereas the ordinal-frequency function yields the reported frequency estimate. For drug $d_{i}$ and side effect $e_{j}$, the pair representation is

(12) $$\phi_{ij}=[h_{d_{i}},h_{e_{j}},h_{d_{i}}⊙h_{e_{j}},|h_{d_{i}}-h_{e_{j}}\left|,{\tilde{s}}_{ij}^{D,0}\right].$$

The association logit, ordinal-frequency estimate, and prevalence-enriched prioritization score are defined as

(13) $$\begin{array}{cc} a_{ij} & =\alpha_{b}{\tilde{s}}_{ij}^{D,0}+\alpha_{r}f_{a}\left(\phi_{ij}\right),\\ {\hat{f}}_{ij} & =f_{f}\left(\phi_{ij}\right)+\beta \left(b_{d_{i}}+b_{e_{j}}\right)+\mu ,\\ {\hat{s}}_{ij}^{D} & =\eta a_{ij}+\left(1-\eta \right)f_{f}\left(\phi_{ij}\right)+\omega_{p}z\left(p_{e_{j}}\right), \end{array}$$

Here, $f_{a}$ and $f_{f}$ are multilayer perceptrons; $b_{d_{i}}$ and $b_{e_{j}}$ are learned drug- and side-effect-specific bias terms, respectively; μ is the mean observed frequency in the training fold; and $z(p_{e_{j}})$ is the within-fold standardized annotation prevalence of side effect $e_{j}$.

### 4.4. Learning Objective and Score Integration

CAFNet-D was optimized jointly for association classification, frequency regression, and ranking:

(14) $$L_{D}=\lambda_{a}L_{\mathrm{BCE}}\left(a,y\right)+\lambda_{f}L_{\mathrm{freq}}\left(\hat{f},S\right)+\lambda_{r}\left\{L_{\mathrm{BPR}}\left({\hat{s}}^{D},y\right)+\lambda_{\ell }L_{\mathrm{ListNet}}\left({\hat{s}}^{D},y\right)\right\},$$

where $y_{ij}=I\left[S_{ij}>0\right]$ is the binary relevance indicator. The Bayesian personalized ranking (BPR) term is computed from positive–unknown pairs sampled according to *y*, whereas the ListNet target distribution is constructed from the same binary relevance labels [15,25]. The ordinal label values, rather than their binarized relevance indicators, enter only the frequency term:

(15) $$L_{\mathrm{freq}}=\frac{1}{\left|P\right|}\sum_{\left(i,j\right):S_{ij}>0}\ell_{\mathrm{Huber}}\left({\hat{f}}_{ij},S_{ij}\right)+\frac{\lambda_{0}}{\left|Z\right|}\sum_{\left(i,j\right):S_{ij}=0}\ell_{\mathrm{Huber}}\left({\hat{f}}_{ij},\epsilon \right),$$

where $\ell_{\mathrm{Huber}}$ denotes the Huber loss [26], and *P* and *Z* denote the sets of non-zero and unobserved training-fold entries, respectively. Entries in *Z* contribute only to the down-weighted low-frequency regularization term.

Drug identities were indexed globally across folds. Drug- and side-effect-specific bias parameters were initialized to zero and updated only from entries in the corresponding training fold. In drug-disjoint evaluation, $b_{d_{i}}=0$ for each held-out drug, whereas $b_{e_{j}}$ and μ were estimated from the training fold. The frequency estimate for a held-out drug included the learned side-effect bias and training-fold mean, but no learned drug-specific bias. Neither bias term contributed to the prioritization score ${\hat{s}}_{ij}^{D}$.

CAFNet was fitted independently under a pointwise matrix-reconstruction objective. For fold *q*, let $E_{C}^{\left(q\right)}$ denote the entries included in CAFNet fitting. Under warm-start evaluation, $E_{C}^{\left(q\right)}=\left\{\left(i,j\right):M_{ij}^{\left(q\right)}=1\right\}$, where $M^{\left(q\right)}$ is the fold-specific training mask; under drug-disjoint evaluation, $E_{C}^{\left(q\right)}=I_{\mathrm{tr}}^{\left(q\right)}\times J$, where $I_{\mathrm{tr}}^{\left(q\right)}$ is the set of training drugs and J is the complete side-effect set. Defining $P_{C}^{\left(q\right)}=\left\{\left(i,j\right)\in E_{C}^{\left(q\right)}:S_{ij}>0\right\}$ and $Z_{C}^{\left(q\right)}=\left\{\left(i,j\right)\in E_{C}^{\left(q\right)}:S_{ij}=0\right\}$, CAFNet minimized

(16) $$L_{C}=\sum_{\left(i,j\right)\in P_{C}^{\left(q\right)}}{\left({\hat{s}}_{ij}^{C}-S_{ij}\right)}^{2}+\lambda_{0}\sum_{\left(i,j\right)\in Z_{C}^{\left(q\right)}}{\left({\hat{s}}_{ij}^{C}-\epsilon \right)}^{2}.$$

The training mask excluded warm-start test entries, whereas drug-disjoint fitting excluded all entries for held-out drugs. Eligible zero-valued entries served as unlabeled background with target ϵ=0.5 and loss weight $\lambda_{0}=0.03$; they were not treated as observed frequency labels or confirmed negative associations. The CAFNet objective did not include association-classification, Huber, BPR, or ListNet losses.

The following values were fixed: η=0.3, $\omega_{p}=0.1$, $\alpha_{b}=\alpha_{r}=\beta =1$, $\lambda_{a}=\lambda_{f}=1$, $\lambda_{r}=0.05$, $\lambda_{\ell }=0.1$, $\lambda_{0}=0.03$, and ϵ=0.5. Annotation prevalence was standardized within each training fold. The BCE class weight equaled the mini-batch ratio of negative to positive entries and was capped at 20. Each drug contributed at most 32 positive–unknown pairs to the BPR term.

The final CAFNet-DG prioritization score is a weighted combination of the CAFNet-D score and the CAFNet graph-interaction score:

(17) $${\hat{s}}_{ij}^{DG}=\rho {\hat{s}}_{ij}^{D}+\left(1-\rho \right){\hat{s}}_{ij}^{C}.$$

For the same-architecture ensemble control, independently fitted CAFNet-D scores were combined without introducing CAFNet predictions. Let ${\hat{s}}_{ij}^{D,r}$ denote the CAFNet-D score from training seed *r*. The equal-weight control was defined as

(18) $${\hat{s}}_{ij}^{\mathrm{DEns}50(3,42)}=0.5{\hat{s}}_{ij}^{D,3}+0.5{\hat{s}}_{ij}^{D,42}.$$

The control used the same held-out folds, candidate sets, metric definitions, and score-averaging procedure as the CAFNet-DG comparison.

CAFNet-D and CAFNet were fitted independently within each data partition. The integration coefficient ρ=0.6 was retained after an exploratory post hoc sweep of stored fold-level predictions from the same 10 drug-disjoint benchmark folds and was then applied uniformly. For ordinal-frequency evaluation, CAFNet-DG retained the CAFNet-D estimate ${\hat{f}}_{ij}$. CAFNet and CAFNet-D were optimized with Adam using the settings in Table 7 [27]. The coefficient and score-normalization analyses in Supplementary Tables S6 and S7 should therefore be interpreted as post hoc sensitivity analyses rather than nested hyperparameter selection.

All CAFNet-family models were trained for a fixed 100 epochs, and predictions from the final epoch were used for evaluation. Early stopping and validation-based checkpoint selection were not applied. The held-out fold was not used for fold-specific epoch selection or checkpoint selection.

### 4.5. Evaluation Design and Statistical Analysis

Internal validation design

All primary analyses used identical 10-fold partitions across models. Warm-start evaluation withheld observed drug–side-effect entries while retaining the corresponding drugs and side effects in the training data. Drug-disjoint evaluation withheld entire drug rows. Scaffold-disjoint evaluation grouped drugs by Bemis–Murcko frameworks; canonical-SMILES groups were used for acyclic compounds [28]. Extended-connectivity fingerprints were used to assess structural similarity across scaffold partitions [29]. All frequency-derived preprocessing and model fitting were confined to the corresponding training fold. In warm-start evaluation, only training-fold entries contributed to loss computation and pair sampling. The same benchmark partitions had been examined during earlier model development and exploratory analyses. Therefore, although the held-out entries in each fold were excluded from that fold’s preprocessing and model fitting, the resulting estimates do not constitute fully untouched outer-test performance under nested validation. They are reported as held-out-fold benchmark results obtained after adaptive development on the same benchmark.

Evaluation metrics

For ADR prioritization, non-zero labels defined relevant associations, whereas unobserved entries served as background candidates. The evaluation metrics were mean average precision (mAP), area under the receiver operating characteristic curve (AUROC), area under the precision–recall curve (AUPR), normalized discounted cumulative gain at 10 (nDCG@10), precision at *k* (P@*k*), and recall at *k* (R@*k*). Within each outer fold, AUROC and AUPR were calculated by pooling all eligible drug–side-effect candidate pairs. AP, nDCG@10, P@*k*, and R@*k* were calculated per drug and macro-averaged across drugs with at least one held-out relevant association. Drugs without a positive candidate were excluded from the per-drug summaries; their eligible negative candidates were retained in the pooled AUROC and AUPR calculations. Pooled metrics were undefined for folds containing fewer than two relevance classes. For nDCG@10, P@*k*, and R@*k*, cutoffs exceeding the number of available candidates were truncated accordingly. Warm-start candidate lists excluded associations visible during training, whereas drug-disjoint evaluation considered all 994 side effects for each held-out drug. Each metric was calculated separately within each outer fold and summarized as the arithmetic mean and sample standard deviation across the 10 folds; predictions were not pooled across folds. Ordinal agreement on held-out non-zero labels was assessed using Spearman correlation, root mean squared error (RMSE), mean absolute error (MAE), quadratic-weighted kappa (QWK), and within-one-class accuracy (Supplementary Table S10) [30,31]. Continuous predictions were used to calculate Spearman correlation, RMSE, and MAE. QWK and within-one-class accuracy were calculated after predictions were clipped to [1,5] and rounded to the nearest ordinal label.

Ablation and annotation-prevalence analyses

The staged drug-disjoint ablation evaluated four formulations: the shared-output CAFNet model; task-specific association and ordinal-frequency functions without ranking or prevalence terms; the same task-specific formulation with BPR and ListNet supervision; and the complete CAFNet-D formulation with drug- and side-effect-specific biases and the prevalence term. Each variant was fitted independently using the same drug-disjoint partitions. The influence of annotation prevalence was examined through a non-personalized prevalence ranking; removal of the 10, 20, 50, and 100 most frequently annotated side effects within each training fold; fold-specific prevalence tertiles; same-drug background controls matched by prevalence bin; and per-drug score differences. Separate component-removal analyses omitted either the association-specific term or the bias and prevalence terms.

Same-architecture ensemble control

The D-Ens50 control defined above was evaluated under the drug-disjoint ADR-ranking setting with the seven primary ranking metrics. Because it combines ranking scores only, no ordinal-frequency output was assigned to the ensemble. Additional ensemble sensitivity analyses are reported in Supplementary Table S12.

External evaluation

External ranking was evaluated using CT-ADE-PT clinical-trial outcomes [32] and OFFSIDES associations [4]. Drug mappings required exact canonical structures, and adverse-event mappings required exact MedDRA preferred terms; pairs already present in the SIDER-derived matrix were excluded. CT-ADE ranking was restricted to events reported at a frequency of at least 1% and evaluated against both the mapped candidate background and same-drug controls sampled at a 1:5 ratio from matched prevalence bins. For ordinal transfer, at-risk-weighted CT-ADE proportions were mapped to the five SIDER frequency categories. Clipped identity, affine, and isotonic calibration functions were selected through five-fold grouped validation of SIDER out-of-fold predictions and subsequently evaluated on CT-ADE. OFFSIDES evaluation used up to five same-drug background controls from matched or adjacent prevalence bins. Additional analyses using OnSIDES v3.1.1 and FDA potential-signal tables are reported in Supplementary Table S1 [33,34].

Comparator methods

CAFNet-DG was compared with CAFNet, CAFNet-D, A3Net, random forest, XGBoost, HSTrans, and DSGAT. Random forest and XGBoost provided non-neural reference methods [35,36]. DSGAT represents graph-based drug and side-effect information, whereas HSTrans models homogeneous drug and side-effect substructures [37,38]. HSTrans side-effect representations were reconstructed within each training fold. DSGAT used the same partitions and a side-effect graph constructed specifically from the corresponding training fold. Annotation prevalence provided a non-personalized control. HMMF was evaluated using regenerated PubMedBERT representations because the original KV-PLM representations were unavailable. Because this comparison used different input representations, its results are reported in Supplementary File S1.

Statistical analysis

Paired comparisons at the fold or drug level used two-sided Wilcoxon signed-rank tests, with Holm adjustment within each prespecified comparator–analysis family [39,40]. Ranking and ordinal-frequency measures were treated as separate families. For prevalence-removal analyses, correction was applied across the four removal thresholds within each comparator–metric combination. Statistical testing of the staged ablation was restricted to adjacent-stage comparisons. For the CAFNet-DG versus D-Ens50 comparison, Holm adjustment was applied jointly across the seven fold-level ranking metrics. These family-wise adjustments were not intended to provide experiment-wide error control across all analyses reported in the study. Because the benchmark folds had overlapping training sets, fold-level comparisons characterize variation across partitions rather than independent experimental replication. Drug-level analyses likewise characterize heterogeneity among held-out drugs rather than independent retraining experiments. The reported tests are conditional on the analyzed model specifications. Exact partitioning procedures, comparator settings, and additional ensemble sensitivity analyses are described in Supplementary Tables S12–S14.

## 5. Conclusions

CAFNet-D provides distinct prediction functions for ADR prioritization and ordinal-frequency estimation, and this task-specific formulation produced the largest joint improvement in the staged drug-disjoint analysis. Aggregate ranking performance was strongly associated with side-effect annotation prevalence. CAFNet-DG adds score integration by combining the CAFNet-D prioritization score with an independently fitted CAFNet graph-interaction score; the matched same-architecture control did not show an advantage for this integration step beyond same-architecture averaging. Scaffold-disjoint and independent-data evaluations showed only modest transfer, and ordinal-frequency agreement on CT-ADE remained limited. Improvements in sparse ADR ranking should therefore be interpreted by distinguishing task-specific output effects from annotation-prevalence and model-averaging effects. The principal method-level contribution is the CAFNet-D task-specific formulation, evaluated together with prevalence-aware analyses; CAFNet-DG supplies the score-integration layer examined under the matched control.

## Figures and Tables

**Table 1:** ADR prioritization under matched 10-fold warm-start and drug-disjoint evaluation. Values are reported as mean ± standard deviation. Boldface denotes the best value among models evaluated with matched inputs within each scenario and measure. ADR, adverse drug reaction; mAP, mean average precision; AUROC, area under the receiver operating characteristic curve; AUPR, area under the precision–recall curve; nDCG, normalized discounted cumulative gain; P, precision; R, recall.

Scenario	Model	mAP	AUROC	AUPR	nDCG@10	P@1	P@15	R@15
Warm	CAFNet	0.207±0.014	0.913±0.003	0.088±0.006	0.261±0.018	0.220±0.023	0.115±0.005	0.408±0.010
Warm	A3Net	0.203±0.026	0.910±0.007	0.080±0.013	0.255±0.034	0.211±0.040	0.113±0.010	0.391±0.036
Warm	RF	0.047±0.003	0.836±0.002	0.022±0.000	0.057±0.005	0.025±0.007	0.027±0.002	0.104±0.010
Warm	XGBoost	0.059±0.003	0.820±0.003	0.027±0.002	0.083±0.005	0.007±0.004	0.034±0.002	0.127±0.007
Warm	HSTrans	0.186±0.009	0.893±0.003	0.074±0.008	0.244±0.011	0.246±0.025	0.103±0.002	0.330±0.010
Warm	DSGAT	0.181±0.018	0.867±0.003	0.059±0.005	0.240±0.021	0.238±0.073	0.102±0.004	0.334±0.014
Warm	CAFNet-D	0.236±0.007	0.909±0.004	0.110±0.006	0.303±0.009	0.329±0.018	0.123±0.002	0.386±0.009
Warm	CAFNet-DG	0.258±0.007	0.926±0.003	0.126±0.005	0.325±0.009	0.343±0.018	0.131±0.003	0.429±0.010
Drug-disjoint	CAFNet	0.340±0.024	0.796±0.015	0.252±0.030	0.492±0.043	0.511±0.087	0.453±0.037	0.230±0.019
Drug-disjoint	A3Net	0.333±0.028	0.795±0.027	0.239±0.034	0.478±0.059	0.484±0.074	0.442±0.050	0.225±0.022
Drug-disjoint	RF	0.197±0.016	0.755±0.024	0.158±0.025	0.342±0.028	0.277±0.075	0.262±0.027	0.130±0.017
Drug-disjoint	XGBoost	0.178±0.016	0.744±0.024	0.145±0.021	0.306±0.025	0.023±0.030	0.229±0.023	0.122±0.019
Drug-disjoint	HSTrans	0.374±0.024	0.802±0.026	0.256±0.023	0.578±0.042	0.659±0.067	0.490±0.041	0.251±0.024
Drug-disjoint	DSGAT	0.389±0.026	0.822±0.019	0.269±0.034	0.596±0.042	0.683±0.064	0.493±0.047	0.253±0.027
Drug-disjoint	CAFNet-D	0.405±0.024	0.818±0.023	0.299±0.032	0.623±0.040	0.707±0.062	0.527±0.043	0.274±0.021
Drug-disjoint	CAFNet-DG	0.413±0.025	0.826±0.021	0.307±0.033	0.626±0.043	0.713±0.066	0.526±0.043	0.273±0.019

**Table 2:** Influence of side-effect annotation prevalence under drug-disjoint evaluation. mAP and nDCG@10 are averaged across drugs. Low-, middle-, and high-prevalence AP values are based on tertiles defined by the number of drugs with a positive annotation for each side effect, calculated exclusively within each training fold. Matched-control metrics compare positive pairs with same-drug background candidates matched by prevalence bin. Boldface denotes the highest displayed value; all ties at the reported precision are marked.

Model	mAP	nDCG@10	High-Prev. AP	Middle-Prev. AP	Low-Prev. AP	Matched AUROC	Matched AUPR
CAFNet	0.340	0.492	0.423	0.122	0.072	0.656	0.683
Annotation prevalence	0.410	0.626	0.487	0.079	0.028	0.623	0.684
CAFNet-D	0.405	0.623	0.488	0.111	0.058	0.662	0.703
CAFNet-DG	0.413	0.626	0.487	0.131	0.079	0.662	0.708

**Table 3:** Same-architecture ensemble control under 10-fold drug-disjoint evaluation. CAFNet-DG denotes $0.6D_{3}+0.4C_{\mathrm{ref}}$, and D-Ens50 denotes the equal-weight control $0.5D_{3}+0.5D_{42}$. Values are mean ± standard deviation across folds. Differences were calculated from the full-precision fold-level values and may not equal subtraction of the displayed rounded means. Two-sided paired Wilcoxon signed-rank *p*-values were adjusted by the Holm procedure across the seven ranking metrics within this comparison.

Metric	CAFNet-DG	D-Ens50 (Seeds 3/42)	DG − D-Ens50	Holm-Adjusted *p*
mAP	0.4134±0.0248	0.4124±0.0243	+0.00095	0.8008
AUROC	0.8257±0.0210	0.8206±0.0251	+0.00518	0.1914
AUPR	0.3066±0.0335	0.3050±0.0317	+0.00167	1.0000
nDCG@10	0.6262±0.0434	0.6259±0.0436	+0.00035	1.0000
P@1	0.7133±0.0660	0.7027±0.0677	+0.01067	1.0000
P@15	0.5265±0.0432	0.5301±0.0415	−0.00364	0.8525
R@15	0.2727±0.0192	0.2761±0.0210	−0.00342	0.2930

**Table 4:** Ordinal-frequency estimation under warm-start and drug-disjoint evaluation. Values are reported as mean ± standard deviation across 10 folds. Boldface denotes the best value within each scenario and measure. Because CAFNet-DG uses the CAFNet-D frequency estimate, a single row represents both models. QWK, quadratic-weighted kappa; Within-1, proportion of predictions within one ordinal class of the observed label.

Scenario	Model	Spearman	RMSE	MAE	QWK	Within-1
Warm	CAFNet	0.525±0.020	1.220±0.027	0.891±0.027	0.426±0.024	0.822±0.009
Warm	A3Net	0.523±0.029	1.189±0.039	0.865±0.033	0.434±0.027	0.832±0.011
Warm	RF	0.319±0.018	1.533±0.020	1.194±0.020	0.209±0.014	0.693±0.010
Warm	XGBoost	0.310±0.012	1.689±0.012	1.478±0.011	0.096±0.007	0.529±0.006
Warm	HSTrans	0.384±0.029	1.337±0.177	0.986±0.156	0.290±0.042	0.787±0.060
Warm	DSGAT	0.437±0.011	1.267±0.091	0.985±0.092	0.306±0.037	0.777±0.034
Warm	CAFNet-D/CAFNet-DG (frequency)	0.705±0.010	0.710±0.021	0.506±0.011	0.640±0.021	0.956±0.005
Drug-disjoint	CAFNet	0.386±0.054	1.754±0.075	1.394±0.066	0.238±0.034	0.621±0.034
Drug-disjoint	A3Net	0.392±0.052	1.712±0.076	1.356±0.084	0.242±0.034	0.635±0.035
Drug-disjoint	RF	0.360±0.052	1.874±0.092	1.609±0.086	0.149±0.023	0.496±0.032
Drug-disjoint	XGBoost	0.347±0.045	1.857±0.097	1.667±0.103	0.094±0.018	0.420±0.050
Drug-disjoint	HSTrans	0.363±0.058	1.829±0.206	1.465±0.202	0.210±0.048	0.613±0.074
Drug-disjoint	DSGAT	0.438±0.062	1.405±0.096	1.143±0.111	0.263±0.046	0.706±0.050
Drug-disjoint	CAFNet-D/CAFNet-DG (frequency)	0.464±0.037	1.165±0.097	0.829±0.070	0.377±0.039	0.841±0.030

**Table 5:** Evaluation on independent CT-ADE-PT data after excluding pairs present in the SIDER-derived matrix. Pooled metrics were calculated over 28,874 candidate pairs; per-drug values are reported as mean ± standard deviation across 29 mapped drugs. Annotation prevalence served as a non-personalized control.

Model	Pooled AUROC	Pooled AUPR	Per-Drug AUROC	Per-Drug AUPR
CAFNet	0.712	0.169	0.786±0.145	0.211±0.175
CAFNet-D	0.748	0.190	0.830±0.115	0.270±0.236
CAFNet-DG	0.754	0.199	0.836±0.107	0.273±0.240
Annotation prevalence	0.741	0.170	0.812±0.106	0.239±0.181

**Table 6:** Distribution of frequency labels for drug–side-effect pairs in the SIDER-derived dataset.

Frequency Label	Frequency Category	Pair Count	Proportion (%)
0	Unknown	708,429	95.03
1	Very rare	1190	0.16
2	Rare	4186	0.56
3	Infrequent	9978	1.34
4	Frequent	17,594	2.36
5	Very frequent	4123	0.55

**Table 7:** Architectural and parameter-estimation settings for CAFNet, CAFNet-D, and CAFNet-DG.

Element	Specification
Input dimensions	109-dimensional atom-feature vectors; 243-dimensional binary side-effect attribute vectors
GAT encoders	Three layers per modality; 10/10/1 attention heads; 200-dimensional output representations; max pooling over drug atoms; node-level side-effect representations retained
Cross-attention	Bidirectional projected dot-product attention; fixed scaling denominator τ=5
Optimization	Adam optimizer; learning rate $4\times 10^{-4}$; weight decay $10^{-3}$
Training schedule	Fixed 100-epoch schedule; final-epoch evaluation; no early stopping; mini-batch size of 10
Side-effect graph	Cosine-similarity *k*-nearest-neighbor graph; k=10 for warm-start evaluation and k=5 for disjoint evaluation
Score integration	Scores from separately fitted CAFNet and CAFNet-D models; ρ=0.6 selected after a post hoc benchmark-fold sweep and then applied uniformly

## Data Availability

Source code, processed benchmark data, fixed partitions, and analysis outputs are available at https://github.com/sskxyc/CAFNet-DG (accessed on 23 September 2026). Original CT-ADE, OnSIDES, FDA, and OFFSIDES resources are available from their respective providers. Derived mappings not included in the public release are available from the corresponding author upon reasonable request.

## Supplementary Materials

The following supporting information can be downloaded at: https://www.mdpi.com/article/10.3390/ijms27198685/s1.

## Abbreviations

The following abbreviations are used in this manuscript:

ADR	Adverse drug reaction
AUPR	Area under the precision–recall curve
AUROC	Area under the receiver operating characteristic curve
BCE	Binary cross-entropy
BPR	Bayesian personalized ranking
CT-ADE	Clinical Trial Adverse Drug Event
FDA	U.S. Food and Drug Administration
GAT	Graph attention network
MAE	Mean absolute error
mAP	Mean average precision
MLP	Multilayer perceptron
nDCG	Normalized discounted cumulative gain
OFFSIDES	Off-label side-effect resource
QWK	Quadratic-weighted kappa
RF	Random forest
RMSE	Root mean squared error
SIDER	Side Effect Resource
SMILES	Simplified Molecular Input Line Entry System
