# Supplementary material for: CAFNet-DG: Task-Specific Modeling and Prevalence-Aware Evaluation for Adverse Drug Reaction Prioritization with Ordinal-Frequency Estimation
Source: Int J Mol Sci. 2026 Sep 28;27(19):8685. doi: 10.3390/ijms27198685 (PMC13665361; doi:10.3390/ijms27198685)
Supplement: Supplementary file 1 [file ijms-27-08685-s001.zip › ijms-4563310-File S1.pdf]

Supplementary Materials for "CAFNet-DG: Task-Specific Modeling and Prevalence-Aware Evaluation for Adverse Drug Reaction Prioritization with Ordinal Frequency Estimation"

Wen Li, Zhiyu Xu, Xiangqiong Wu, Ju Xiang and Xiaojie Zhang*

1 Data Partitions and Evaluation

Primary comparisons followed prespecified warm-start or drug-disjoint partitions. In each fold, the frequency-derived side-effect graph and HSTrans side-effect representations were constructed from training data only. Drug-disjoint evaluation withheld complete drug rows, whereas warm-start evaluation excluded validation and test coordinates from optimization and sampling. Metrics and paired tests were calculated from the corresponding held-out predictions.

2 External Evaluation

CT-ADE-PT provided the clinical-trial outcomes used for the principal external evaluation. Exact canonical-structure mapping yielded 31 benchmark drugs and 254 trial groups, and exact MedDRA preferred-term mapping covered 982 of 994 side-effect terms. After SIDER-observed pairs were removed, the prioritization analysis comprised 1,336 adverse-event pairs reported at a frequency of at least 1% across 29 drugs and 27,538 background candidates. The main text reports the pooled and per-drug results. In per-drug paired Wilcoxon tests, CAFNet-DG had higher AUROC ($p_{\mathrm{Holm}} = 0.0216$) and AUPR ($p_{\mathrm{Holm}} = 0.00110$) than CAFNet. Differences from CAFNet-D and annotation prevalence were not significant.

For ordinal-frequency transfer, at-risk-weighted trial proportions were mapped to the five SIDER frequency bands, yielding 2,781 non-SIDER pairs across 30 drugs. The raw CAFNet-D output did not outperform the training-side-effect mean baseline (Table S1). After Holm correction, it performed significantly worse for within-one accuracy, exact accuracy, RMSE, and MAE; QWK and Spearman did not differ significantly.

A post-hoc analysis compared clipped identity, affine, and isotonic calibration using grouped validation of SIDER out-of-fold predictions. Isotonic calibration had the lowest validation RMSE and was selected without access to CT-ADE labels. It reduced CT-ADE scale error but did not significantly outperform the training-side-effect mean baseline in per-drug comparisons and yielded lower QWK. This pattern is consistent with scale mismatch but does not establish transferable ordinal discrimination.

Table S1: CT-ADE external ordinal-frequency and SIDER-only post-hoc calibration analysis. Lower is better for RMSE and MAE; higher is better otherwise. Bold indicates the numerical best value and does not imply a significant per-drug difference.

Scoring approach	QWK	Within-one	Exact accuracy	Spearman	RMSE	MAE
CAFNet-D frequency (raw)	**0.288**	0.755	0.269	0.347	1.391	1.126
CAFNet-D + isotonic calibration	0.209	**0.924**	0.391	0.344	**0.867**	**0.697**
Training-side-effect mean baseline	0.261	0.894	**0.405**	**0.360**	0.901	0.727

The OnSIDES v3.1.1 mapping contained 91 high-confidence non-SIDER positive pairs across 27 drugs and 455 positive–control pairs. CAFNet-DG achieved an AUROC of 0.540, an AUPR of 0.552, and a tie-aware concordance of 0.602, without significant paired superiority over the learned component scores. FDA AEMS/FAERS quarterly potential-signal tables from 2023–2026 yielded 53 mapped non-SIDER signals across 39 drugs and 265 positive–control pairs. CAFNet-DG achieved an AUROC of 0.547, an AUPR of 0.538, and a concordance of 0.555, again without significant paired superiority. FDA entries were treated as potential safety signals rather than confirmed causal ADR labels.

3 Prevalence-Matched Evaluation on OFFSIDES

We also evaluated CAFNet-DG on OFFSIDES-derived external positive drug–side-effect pairs. After mapping OFFSIDES pairs to the 750-drug/994-side-effect benchmark, we obtained 35,683 unique mapped positive pairs

covering 463 drugs and 540 side effects, excluding pairs already observed in the SIDER-derived matrix. For each positive pair, up to five same-drug unlabeled controls were sampled from matched or adjacent training-fold side-effect prevalence bins. This produced 178,415 matched positive–control pairs with a median absolute prevalence difference of 0.0015.

Table S2: OFFSIDES prevalence-matched external analysis. Annotation prevalence is a non-personalized control.

Scoring approach	AUROC	AUPR	Positive-over-control rate
CAFNet	0.5167	0.5171	0.5221
CAFNet-D	0.5171	0.5156	0.5299
CAFNet-DG	**0.5183**	**0.5175**	**0.5310**
Annotation prevalence	0.5023	0.5058	0.3955

CAFNet-DG attained the highest pooled AUROC, AUPR, and positive-over-control rate in this matched setting. Per-drug Wilcoxon tests over the 463 mapped drugs showed small but significant improvements of CAFNet-DG over CAFNet-D in external AUROC (0.5218 versus 0.5205, Holm-adjusted $p = 0.00496$) and external AUPR (0.5307 versus 0.5291, Holm-adjusted $p = 3.29 \times 10^{-4}$). CAFNet-DG was also significantly higher than annotation prevalence on all three reported per-drug metrics. The effect size was small and indicates a modest score-integration difference under the matched-control design; it does not establish architecture-specific complementarity or constitute clinical validation.

4 Component and Annotation-Prevalence Analyses

Component-removal experiments were evaluated under the same 10 drug-disjoint folds. Removing the bias and annotation-prevalence terms reduced aggregate ranking performance, whereas removing the association-specific term produced only small numerical changes.

Table S3: Component-removal analysis under drug-disjoint evaluation. Values are 10-fold means.

Configuration	mAP	nDCG@10	Low-prev. AP	Matched AUPR
CAFNet-D	0.405	0.623	0.058	0.703
w/o association-specific term	0.407	0.626	0.060	0.704
w/o bias/annotation-prevalence terms	0.382	0.594	0.058	0.698

The prevalence and bias terms contributed primarily to aggregate retrieval. They did not improve low-prevalence AP, supporting the distinction between prevalence-enriched ranking and recovery of less frequently annotated ADRs.

CAFNet-D had higher mAP and nDCG@10 than CAFNet and outperformed annotation prevalence in the prevalence-matched control test. Its low- and middle-prevalence AP values, however, were lower than those of CAFNet. Relative to CAFNet-D, CAFNet-DG increased low-prevalence AP from 0.058 to 0.079, middle-prevalence AP from 0.111 to 0.131, and matched AUPR from 0.703 to 0.708. Low-prevalence AP did not consistently exceed CAFNet across evaluation protocols. Thus, score integration changed prevalence-stratified ranking behavior but did not resolve low-prevalence ADR retrieval; the matched same-architecture controls reported below show that these differences do not establish a unique cross-architecture performance benefit.

5 Ablation of Task-Specific Prediction Components

The staged ablation used the same 10 drug-disjoint folds. The *Task-specific functions* stage replaced the shared output with task-specific association and ordinal-frequency functions without ranking supervision, annotation-prevalence terms, or bias terms. The + *Pairwise/listwise ranking* stage added BPR and ListNet supervision. The final stage added drug- and side-effect-specific bias terms and the training-fold annotation-prevalence term. Each variant was fitted independently, so statistical comparisons were restricted to this ablation series. Spearman correlation, RMSE, and MAE were calculated from continuous scalar predictions. QWK and within-one-class accuracy were calculated after clipping predictions to $[1, 5]$ and rounding them to the nearest ordinal label.

Fold-level comparisons used two-sided Wilcoxon signed-rank tests with Holm adjustment within the ranking and ordinal metric families. Table S5 summarizes the changes most relevant to the conclusions in the main text.

Replacing the shared output with task-specific association and ordinal-frequency functions produced the largest joint change in this ablation series. Ranking supervision alone produced small, nonsignificant changes. Adding the bias and annotation-prevalence terms improved aggregate ranking without improving ordinal-frequency estimates.

Table S4: Staged ablation of task-specific prediction functions under drug-disjoint evaluation. Values are mean ± standard deviation over 10 folds. Comparisons are restricted to this independently trained ablation series. Bold indicates the best mean in each column.

(a) Aggregate ADR prioritization

Stage	mAP	AUROC	AUPR	nDCG@10
CAFNet	0.340 ± 0.024	0.796 ± 0.015	0.252 ± 0.030	0.492 ± 0.043
Task-specific functions	0.382 ± 0.029	0.806 ± 0.024	0.265 ± 0.035	0.591 ± 0.046
+ Pairwise/listwise ranking	0.388 ± 0.022	0.807 ± 0.022	0.263 ± 0.031	0.604 ± 0.037
Full CAFNet-D (ablation series)	0.405 ± 0.024	0.812 ± 0.023	0.292 ± 0.033	0.625 ± 0.046

(b) Top-ranked retrieval

Stage	P@1	P@15	R@15
CAFNet	0.511 ± 0.087	0.453 ± 0.037	0.230 ± 0.019
Task-specific functions	0.671 ± 0.079	0.500 ± 0.046	0.259 ± 0.028
+ Pairwise/listwise ranking	0.680 ± 0.072	0.502 ± 0.039	0.262 ± 0.028
Full CAFNet-D (ablation series)	0.700 ± 0.071	0.525 ± 0.042	0.274 ± 0.020

(c) Ordinal-frequency estimation

Stage	Spearman	RMSE	MAE	QWK	Within-one
CAFNet	0.386 ± 0.054	1.754 ± 0.075	1.394 ± 0.066	0.238 ± 0.034	0.621 ± 0.034
Task-specific functions	0.465 ± 0.038	1.142 ± 0.059	0.818 ± 0.043	0.398 ± 0.041	0.845 ± 0.020
+ Pairwise/listwise ranking	0.471 ± 0.045	1.167 ± 0.082	0.840 ± 0.071	0.391 ± 0.043	0.835 ± 0.027
Full CAFNet-D (ablation series)	0.467 ± 0.045	1.170 ± 0.072	0.836 ± 0.046	0.387 ± 0.036	0.839 ± 0.019

Table S5: Key paired comparisons in the staged ablation. S0 denotes CAFNet, S1 Task-specific functions, S2 the addition of pairwise/listwise ranking, and S3 full CAFNet-D. Values in parentheses are Holm-adjusted Wilcoxon p-values. Positive RMSE changes indicate reduced error.

Comparison	ΔmAP	ΔnDCG@10	RMSE reduction	ΔQWK
S1 vs S0	0.0421 (0.0137)	0.0983 (0.0137)	0.6119 (0.0098)	0.1600 (0.0098)
S2 vs S1	0.0062 (1.0000)	0.0138 (0.5879)	−0.0243 (1.0000)	−0.0069 (1.0000)
S3 vs S2	0.0178 (0.0137)	0.0202 (0.0137)	−0.0030 (1.0000)	−0.0036 (1.0000)

6 Sensitivity to the Integration Coefficient

A post-hoc sensitivity analysis combined the same held-out component predictions using ρ values from 0 to 1. The primary coefficient, $\rho = 0.6$, was retained after exploratory inspection of these cached drug-disjoint predictions and was subsequently frozen for the final evaluations. This analysis should therefore be interpreted as a post-hoc sensitivity analysis rather than nested hyperparameter selection. Aggregate ranking metrics changed little across $\rho = 0.5$–0.8, whereas low-prevalence AP was numerically highest at $\rho = 0.3$.

Table S6: Drug-disjoint sensitivity to the integration coefficient. Values are 10-fold means.

ρ	mAP	AUPR	nDCG@10	Top-100-removed mAP	Low-prev. AP
0.0	0.340	0.252	0.492	0.109	0.076
0.3	0.392	0.288	0.592	0.119	**0.084**
0.5	0.409	0.303	0.621	0.122	0.081
0.6	0.413	0.307	0.626	0.122	0.078
0.7	**0.414**	**0.308**	**0.627**	0.123	0.078
0.8	0.413	**0.308**	0.626	**0.124**	0.073
1.0	0.405	0.299	0.623	0.113	0.063

7 Sensitivity to Score Normalization

The component scores have different numerical scales. We therefore compared raw scores with per-drug z-score and percentile transformations at the fixed coefficient $\rho = 0.6$. No outcome labels were used in these transformations.

Raw-score integration gave the highest mean for all three reported ranking metrics. However, the differences across normalization schemes show that score integration remained sensitive to component scaling.

Table S7: Drug-disjoint sensitivity to score normalization at $\rho = 0.6$. Values are mean $\pm$ standard deviation.

Integration input	mAP	AUPR	nDCG@10
Raw scores	0.413 ± 0.025	0.307 ± 0.033	0.626 ± 0.043
Per-drug z-scores	0.403 ± 0.025	0.268 ± 0.021	0.612 ± 0.043
Per-drug percentiles	0.408 ± 0.025	0.293 ± 0.024	0.618 ± 0.042

8 Per-Drug Variation in Ranking Performance

Per-drug analyses quantified how often score integration changed ranking performance among held-out drugs.

Table S8: Per-drug differences between CAFNet-DG and CAFNet-D under drug-disjoint evaluation. Confidence intervals are Wilson 95% intervals for the proportion of drugs with a positive difference. Exact p-values are from two-sided binomial tests against a 50% null. These tests assess the direction, but not the magnitude, of the per-drug differences.

Metric	Mean difference	Median difference	Positive n/N	Positive fraction	95% CI	Exact p
AP	0.0084	0.0054	446/750	0.595	0.559–0.629	2.43×10^{-7}
Low-prevalence AP	0.0149	0.0003	254/442	0.575	0.528–0.620	0.0020
Middle-prevalence AP	0.0213	0.0013	332/545	0.609	0.568–0.649	3.90×10^{-7}
Top-100-removed AP	0.0099	0.0023	458/714	0.641	0.606–0.676	3.66×10^{-14}

Positive per-drug differences occurred more often than expected under a 50% directional null in all four analyses. However, the median differences were small and varied across prevalence strata and removal settings. These results therefore indicate heterogeneous directional improvement rather than a large or uniform per-drug benefit. These comparisons are against a single CAFNet-D fit and should be interpreted together with the matched same-architecture ensemble controls in Tables S12–S14, which do not show a stable advantage of CAFNet-DG over CAFNet-D ensembles.

9 Evaluation after Removal of Frequently Annotated Side Effects

The top-N most frequently annotated side effects were excluded from each candidate set before the ranking metrics were recalculated. This analysis assessed whether between-model differences persisted after removal of the strongest annotation-prevalence signals.

Table S9: Drug-disjoint ranking metrics after removing the top-N most frequently annotated side effects. All nonzero frequency labels were treated as binary relevance. Metrics were macro-averaged over held-out drugs with at least one positive label remaining; the number of evaluable drugs per fold ranged from 74–75, 73–75, 73–75, and 70–74 after removal of the top 10, 20, 50, and 100 side effects, respectively. Values are means across the 10 folds.

Removed	Model	mAP	nDCG@10	P@15	R@15
Top-10	CAFNet	0.2430	0.3508	0.3130	0.1776
Top-10	CAFNet-D	0.2730	0.4173	0.3599	0.2071
Top-10	CAFNet-DG	0.2844	0.4227	0.3638	0.2094
Top-20	CAFNet	0.2006	0.2810	0.2484	0.1525
Top-20	CAFNet-D	0.2251	0.3361	0.2838	0.1751
Top-20	CAFNet-DG	0.2362	0.3417	0.2907	0.1806
Top-50	CAFNet	0.1520	0.2154	0.1760	0.1471
Top-50	CAFNet-D	0.1670	0.2360	0.1879	0.1507
Top-50	CAFNet-DG	0.1820	0.2549	0.2011	0.1686
Top-100	CAFNet	0.1091	0.1453	0.1162	0.1129
Top-100	CAFNet-D	0.1128	0.1426	0.1141	0.1173
Top-100	CAFNet-DG	0.1224	0.1592	0.1263	0.1280

Fold-level comparisons used two-sided Wilcoxon signed-rank tests with Holm adjustment across the four removal thresholds within each comparator–metric family. Relative to CAFNet, CAFNet-DG was significantly higher for all four metrics after top-10, top-20, and top-50 removal. After top-100 removal, the differences remained significant for mAP, nDCG@10, and P@15, whereas R@15 was numerically higher.

Relative to CAFNet-D, CAFNet-DG was significantly higher for mAP after top-10, top-20, and top-50 removal. The differences were also significant for P@15 after top-50 and top-100 removal and for R@15 after top-50 removal. None of the nDCG@10 differences between CAFNet-DG and CAFNet-D remained significant after Holm adjustment.

These results indicate that part of the ranking difference persisted after removal of the most frequently annotated side effects, although the differences from CAFNet-D were metric- and threshold-dependent.

For CAFNet-DG versus CAFNet-D, the Holm-adjusted p-values at top-10, top-20, top-50, and top-100 removal were, respectively, 0.0078, 0.0117, 0.0117, and 0.0645 for mAP; 0.7500, 0.7500, 0.0781, and 0.1934 for nDCG@10; 0.5566, 0.3461, 0.0156, and 0.0293 for P@15; and 0.4922, 0.2520, 0.0391, and 0.2520 for R@15.

10 Sensitivity of the Frequency Output

The unknown-entry target was evaluated in a post-hoc drug-disjoint sensitivity analysis. Changes in λ_0 and ϵ produced substantial differences in ordinal-frequency errors and smaller changes in ordinal agreement.

Table S10: Sensitivity of the CAFNet-D frequency output to the unknown-entry prior. Selected settings are shown; values are mean ± standard deviation over 10 folds. The reference configuration was independently refitted within this sensitivity series and therefore does not duplicate the primary CAFNet-D estimate.

Setting	RMSE	MAE	QWK	Within-one
Reference fit ($\lambda_0 = 0.03, \epsilon = 0.50$)	1.208 ± 0.087	0.861 ± 0.052	0.384 ± 0.050	0.832 ± 0.020
$\lambda_0 = 0.03, \epsilon = 0.75$	1.092 ± 0.132	0.791 ± 0.092	0.389 ± 0.049	0.852 ± 0.042
$\lambda_0 = 0.00, \epsilon = 0.50$	0.869 ± 0.061	0.645 ± 0.042	0.408 ± 0.067	0.915 ± 0.020
$\lambda_0 = 0.10, \epsilon = 0.50$	1.375 ± 0.102	0.985 ± 0.079	0.349 ± 0.040	0.784 ± 0.031

These settings were evaluated without nested validation and were not used for model selection. The variation across settings indicates that ordinal-frequency estimates remained sensitive to the treatment of unknown entries.

11 Scaffold-Disjoint Evaluation

Bemis–Murcko scaffolds were available for 707 drugs, whereas 43 acyclic drugs were assigned canonical-SMILES groups. This produced 578 non-overlapping groups for fixed 10-fold evaluation. Each fold contained 75 test drugs, with no scaffold-group or exact canonical-structure overlap between the training and test sets. CAFNet and CAFNet-D were fitted under matched conditions using the same folds. Their corresponding predictions were combined using $\rho = 0.6$, which had been retained after post-hoc examination of the drug-disjoint benchmark predictions and was transferred unchanged to the scaffold-disjoint evaluation without selection on the scaffold-test folds.

CAFNet-DG had the highest mAP, global AUROC, global AUPR, and Top-100-removed mAP (Table S11). Low- and middle-prevalence differences were not significant, so the scaffold-disjoint results do not establish an advantage for less frequently annotated ADRs.

Table S11: Scaffold-disjoint ranking and prevalence-stratified comparison. Values are mean ± standard deviation over 10 fixed folds. Bold indicates the highest mean.

(a) Aggregate ranking

Model	mAP	Global AUROC	Global AUPR	nDCG@10	Top-100-removed mAP
CAFNet	0.334 ± 0.013	0.788 ± 0.019	0.246 ± 0.016	0.483 ± 0.031	0.105 ± 0.015
CAFNet-D	0.404 ± 0.021	0.809 ± 0.020	0.292 ± 0.019	0.626 ± 0.025	0.104 ± 0.016
CAFNet-DG	0.409 ± 0.020	0.817 ± 0.019	0.298 ± 0.016	0.621 ± 0.027	0.117 ± 0.014

(b) Prevalence-stratified AP

Model	Low-prevalence AP	Middle-prevalence AP	High-prevalence AP
CAFNet	0.067 ± 0.013	0.099 ± 0.012	0.383 ± 0.017
CAFNet-D	0.059 ± 0.019	0.093 ± 0.015	0.453 ± 0.019
CAFNet-DG	0.066 ± 0.020	0.103 ± 0.019	0.456 ± 0.019

12 Software and Computing Environment

Model fitting and evaluation were performed using Python 3.9.21, PyTorch 1.12.1 with CUDA 11.3, PyTorch Geometric 1.7.2, and RDKit 2022.9.4. Numerical analyses used NumPy 1.22.4, pandas 1.5.1, SciPy 1.10.1, and scikit-learn 1.2.1. Experiments were run under Windows 10 using an NVIDIA GeForce RTX 4060 Ti GPU. The complete dependency specification is provided with the accompanying code.

13 Numerical Stability and Mini-Batch Sensitivity

Across three repeated fits of one fixed drug-disjoint fold, mAP ranged from 0.371 to 0.386 and RMSE ranged from 1.064 to 1.187 despite use of the same partition and random seed. This run-to-run variation should be considered when interpreting small numerical differences between models.

To assess sensitivity to inference-batch composition, the accompanying drugs were varied while the target drug was held fixed. Prioritization scores changed by at most 2.064×10^{-3}, rank correlations exceeded 0.999997, and the top-15 candidate set was unchanged. Mini-batch composition therefore had a detectable but negligible effect on the reported rankings.

14 Comparator Context

HSTrans side-effect representations were reconstructed independently within each training fold. DSGAT used the same warm-start and drug-disjoint partitions as the CAFNet-family models, with its side-effect graph reconstructed from training-fold data.

HMMF was treated as a descriptive comparison because the original KV-PLM representations were unavailable. Using regenerated PubMedBERT representations, HMMF attained drug-disjoint mAP of 0.386 ± 0.027, AUPR of 0.348 ± 0.073, RMSE of 0.831 ± 0.074, and QWK of 0.383 ± 0.083. It was excluded from the primary input-matched comparison because the reconstructed text representation differed from that of the original method.

15 Matched Same-Architecture Ensemble Controls

To distinguish cross-architecture score integration from the generic effect of averaging independently fitted predictors, the reference CAFNet-DG ranking score was compared with ensembles formed only from independently fitted CAFNet-D models. The reference configuration is denoted $0.6D_3 + 0.4C_{\text{ref}}$, where D_3 is the CAFNet-D fit used in the reported CAFNet-DG analysis and C_{ref} is the independently fitted CAFNet score source paired with it. Equal-weight CAFNet-D ensembles were evaluated for seed pairs $(3, 42)$, $(3, 2026)$, and $(42, 2026)$, and a weight-matched $0.6D_3 + 0.4D_{42}$ control was evaluated as a sensitivity analysis. All controls used the same drug-disjoint folds, candidate sets, and metric implementation. Only ranking outputs were compared.

Table S12: Matched same-architecture ensemble performance under drug-disjoint evaluation. Values are mean ± standard deviation across 10 folds. Bold is intentionally omitted because the purpose of this table is control analysis rather than model ranking.

Family	Formula	mAP	AUROC	AUPR	nDCG@10	P@1	P@15	R@15
CAFNet-DG	$0.6D_3 + 0.4C_{\text{ref}}$	0.4134 ± 0.0248	0.8257 ± 0.0210	0.3066 ± 0.0335	0.6262 ± 0.0434	0.7133 ± 0.0660	0.5265 ± 0.0432	0.2727 ± 0.0192
D-Ens50	$0.5D_3 + 0.5D_{42}$	0.4124 ± 0.0243	0.8206 ± 0.0251	0.3050 ± 0.0317	0.6259 ± 0.0436	0.7027 ± 0.0677	0.5301 ± 0.0415	0.2761 ± 0.0210
D-Ens50	$0.5D_3 + 0.5D_{2026}$	0.4143 ± 0.0239	0.8234 ± 0.0230	0.3072 ± 0.0336	0.6279 ± 0.0420	0.7160 ± 0.0622	0.5278 ± 0.0416	0.2735 ± 0.0200
D-Ens50	$0.5D_{42} + 0.5D_{2026}$	0.4145 ± 0.0253	0.8169 ± 0.0273	0.3023 ± 0.0355	0.6281 ± 0.0467	0.7067 ± 0.0762	0.5288 ± 0.0424	0.2755 ± 0.0204
D-Ens60	$0.6D_3 + 0.4D_{42}$	0.4122 ± 0.0243	0.8215 ± 0.0247	0.3056 ± 0.0318	0.6261 ± 0.0440	0.7053 ± 0.0693	0.5296 ± 0.0429	0.2753 ± 0.0212

None of the 21 equal-weight fold-level comparisons remained significant after Holm correction. The seven weight-matched $0.6D_3 + 0.4D_{42}$ comparisons led to the same conclusion: CAFNet-DG was numerically higher for mAP, AUROC, AUPR, nDCG@10, and P@1 and lower for P@15 and R@15, but none of these differences remained significant after Holm adjustment. Exact statistics for both the equal-weight and weight-matched controls are reported in Table S13.

The primary D-Ens(3,42) comparison yielded an AP-improvement fraction of $379/750 = 50.5\%$ and an nDCG@10-improvement fraction of $280/750 = 37.3\%$ when ties were counted in the denominator. The AP result was therefore close to a balanced directional split, while nDCG@10 contained many exact ties. No drug-level comparison was significant after Holm adjustment. Taken together, the matched controls show that the observed aggregate CAFNet-DG gain cannot be uniquely attributed to cross-architecture complementarity and is compatible with generic ensemble variance reduction.

Table S13: Fold-level paired comparisons of the reference CAFNet-DG configuration with three equal-weight CAFNet-D ensembles and the weight-matched CAFNet-D ensemble. Δ is CAFNet-DG minus the comparator. Holm correction was applied across the seven metrics within each comparator family.

Comparator	Metric	Δ	Wilcoxon p	Holm-adjusted p
D-Ens(3,42)	mAP	0.00095	0.1602	0.8008
D-Ens(3,42)	AUROC	0.00518	0.0273	0.1914
D-Ens(3,42)	AUPR	0.00167	0.4316	1.0000
D-Ens(3,42)	nDCG@10	0.00035	0.8457	1.0000
D-Ens(3,42)	P@1	0.01067	0.3657	1.0000
D-Ens(3,42)	P@15	-0.00364	0.2131	0.8525
D-Ens(3,42)	R@15	-0.00342	0.0488	0.2930
D-Ens(3,2026)	mAP	-0.00091	0.6953	1.0000
D-Ens(3,2026)	AUROC	0.00230	0.5566	1.0000
D-Ens(3,2026)	AUPR	-0.00054	0.7695	1.0000
D-Ens(3,2026)	nDCG@10	-0.00165	0.7695	1.0000
D-Ens(3,2026)	P@1	-0.00267	0.9165	1.0000
D-Ens(3,2026)	P@15	-0.00133	0.9219	1.0000
D-Ens(3,2026)	R@15	-0.00079	0.9219	1.0000
D-Ens(42,2026)	mAP	-0.00113	1.0000	1.0000
D-Ens(42,2026)	AUROC	0.00882	0.1602	1.0000
D-Ens(42,2026)	AUPR	0.00434	0.3223	1.0000
D-Ens(42,2026)	nDCG@10	-0.00184	0.6250	1.0000
D-Ens(42,2026)	P@1	0.00667	0.7661	1.0000
D-Ens(42,2026)	P@15	-0.00231	0.6953	1.0000
D-Ens(42,2026)	R@15	-0.00280	0.0645	0.3867
D-Ens60(3,42)	mAP	0.00117	0.1602	0.8008
D-Ens60(3,42)	AUROC	0.00427	0.0273	0.1914
D-Ens60(3,42)	AUPR	0.00105	0.4922	1.0000
D-Ens60(3,42)	nDCG@10	0.00009	0.9219	1.0000
D-Ens60(3,42)	P@1	0.00800	0.2359	0.9435
D-Ens60(3,42)	P@15	-0.00311	0.3428	1.0000
D-Ens60(3,42)	R@15	-0.00261	0.0488	0.2930

Table S14: Drug-level comparisons of CAFNet-DG with equal-weight CAFNet-D ensembles. Confidence intervals are 20,000-replicate drug-bootstrap 95% intervals. Permutation p-values are paired drug-level sign-flip tests; the reported Holm-adjusted values are adjusted across the six seed-pair/metric comparisons.

Comparator	Metric	Mean Δ	95% CI low	95% CI high	Positive drugs	Permutation p	Holm p
D-Ens(3,42)	AP	0.000946	-0.001992	0.003928	379/750	0.5278	1.0000
D-Ens(3,42)	nDCG@10	0.000351	-0.005032	0.005557	280/750	0.8955	1.0000
D-Ens(3,2026)	AP	-0.000914	-0.003800	0.002049	367/750	0.5370	1.0000
D-Ens(3,2026)	nDCG@10	-0.001645	-0.006937	0.003534	265/750	0.5376	1.0000
D-Ens(42,2026)	AP	-0.001127	-0.004674	0.002410	380/750	0.5343	1.0000
D-Ens(42,2026)	nDCG@10	-0.001841	-0.007853	0.004063	279/750	0.5453	1.0000
